# Keeping in Mind Its Synchronous Features, Is Sinonasal Inverted Papilloma Always Benign?

**DOI:** 10.7759/cureus.27498

**Published:** 2022-07-31

**Authors:** Mohamed Iliyas Sultan Abdul Kader, Urscilla Jaya Prahaspathiji, Abd Razak Ahmad, Farah Dayana Zahedi

**Affiliations:** 1 Department of Otorhinolaryngology, Head and Neck Surgery, Hospital Melaka, Melaka, MYS; 2 Department of Otorhinolaryngology, Head and Neck Surgery, Faculty of Medicine, Universiti Kebangsaan, Kuala Lumpur, MYS; 3 Department of Otorhinolaryngology, Head and Neck Surgery, Faculty of Medicine, Universiti Kebangsaan Malaysia, Kuala Lumpur, MYS

**Keywords:** synchronous malignancy, tumor imaging, sqamous cell carcinoma, inverted papilloma, paranasal sinuses

## Abstract

Sinonasal inverted papilloma (SNIP) is a rare benign tumor of paranasal sinuses. SNIP is known to be locally aggressive, with high rates of recurrence and a high potential for malignant transformation. We present a patient who presented with right-sided cheek pain and swelling for two weeks. The initial biopsy revealed SNIP. However, postoperative histopathology examination results revealed synchronous squamous cell carcinoma (SCC) with sinonasal inverted papilloma. Although the initial biopsy result showed a benign lesion, the aggressive features such as bony destruction and orbital involvement in computed tomography scan should raise a suspicion of a malignant lesion. Imaging features of SNIP from synchronous SCC are discussed.

## Introduction

Sinonasal inverted papilloma (SNIP) is a benign tumor of the paranasal sinuses. SNIP is a histological subtype of papilloma that originates from Schneiderian mucosa, an ectodermally derived respiratory epithelium. SNIP accounts for the majority of Schneiderian papilloma, and other subtypes include exophytic papilloma and oncocytic papilloma [1]. Despite being benign, SNIP is known to be locally aggressive, with high rates of recurrence and potential malignant transformation.

Synchronous carcinomas are defined as carcinoma that occurs simultaneously or within a six-month period of the primary malignancy, while it's considered metachronous after this period [2]. Five-year overall survival of SNIP with squamous cell carcinoma (SCC) was 70% compared to 59.5% in sinonasal SCC [3,4]. Identification is crucial for appropriate management as a successful treatment increases the overall survival rate.

We present a patient who presented with right maxillary swelling reported to be SNIP; however, in imaging, it shows extensive bony destruction and therefore warranted an open surgery; histopathological examination (HPE) revealed synchronous SCC with SNIP. In this report, we discuss how to differentiate SNIP with synchronous SCC and its management based on literature.

## Case presentation

**Figure 1:**
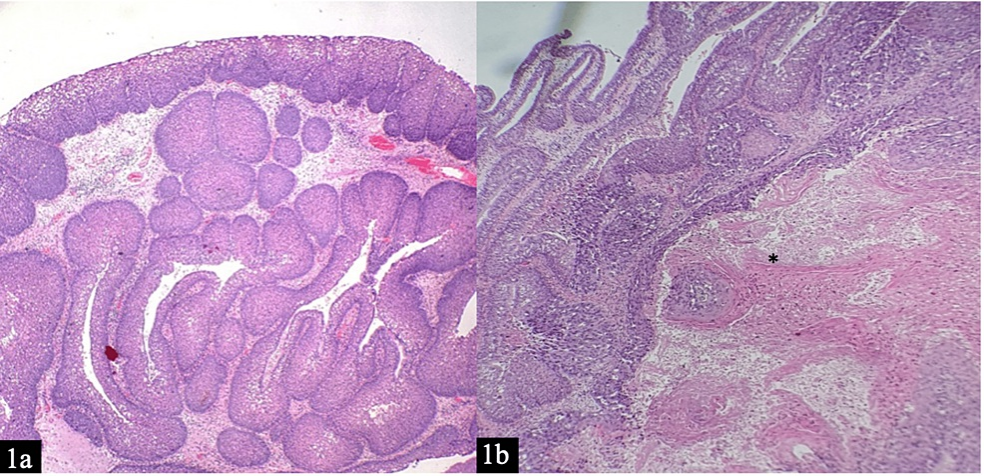
Findings of histopathological examination a: hematoxylin & eosin (H&E) 4X shows inverted papilloma, composed of prominent downward endophytic growth. b: H&E 10X shows inverted papilloma along with adjacent malignant transformation (*).

**Figure 2:**
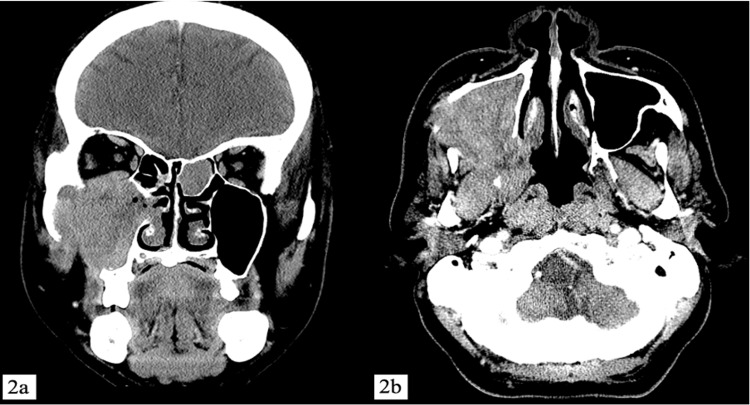
Contrast-enhanced computed tomography (CECT) of paranasal sinuses coronal (a) and axial (b) views CECT shows extensive hypodense mass occupying the whole of the right maxillary sinus with bony destruction and erosion of the right orbital floor, lateral wall of maxilla with extension to cheek.

A 65-year-old man who was a chronic smoker but with no known comorbidities presented with right cheek pain and swelling for two months. The swelling was progressively increasing in size immediately after tooth extraction two weeks prior to the presentation. Nasoendoscopic examination revealed a right pinkish irregular nasal mass arising from the right middle meatus. A biopsy was taken, and a histopathological examination reported SNIP (Figure 1a). Contrast-enhanced computed tomography (CECT) of paranasal sinuses revealed a mass measuring 5.5 x 3.2 cm with extensive bony destruction with intraorbital extension and erosion of the lateral wall of the maxilla with extension to the cheek (Figure 2).

**Figure 3:**
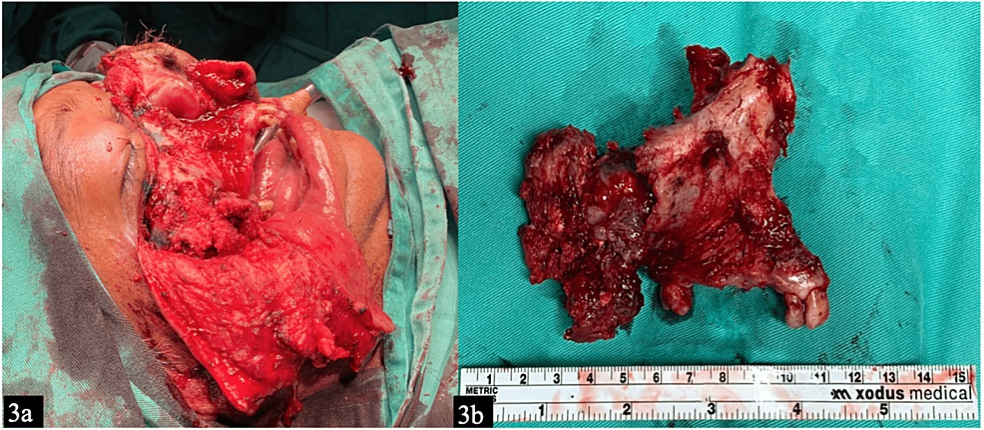
Post Weber-Ferguson incision shows the tumor (a), and the right total maxillectomy along with the tumor sent for histopathological examination (b)

Due to the extensive nature of the swelling, he underwent right total maxillectomy and ethmoidectomy via classic Weber-Ferguson incision (Figure 3a). His orbital floor was reconstructed, and the patient had prosthetic rehabilitation with an obturator. A gross pathological examination (Figure 3b) showed the right maxilla bone with the attached two incisors and one molar tooth with a fungating tumor measuring 52 x 60 x 56 mm arising from the mucosa covering the right maxillary bone.

Microscopic description (Figure 1b) showed inverted papilloma composed of prominent downward endophytic growth of round elongated interconnected squamous and respiratory-type epithelial nests with smooth outer contour displaying adjacent malignant transformation. The malignant cells show moderate pleomorphism with nuclear enlargement, hyperchromatism, vesicular chromatin pattern, and prominent nucleoli. Intercellular bridges, individual cell keratinization with keratin pearl formation. These findings are in keeping with well-differentiated keratinizing squamous cell carcinoma of malignant transformation from an inverted papilloma. The patient was staged T4aN0M0 according to the American Joint Committee on Cancer (AJCC) eighth edition. The patient refused adjuvant radiotherapy despite counseling. He developed recurrence and died of catastrophic bleeding in his house, one year from diagnosis. 

## Discussion

SNIP is a rare tumor of paranasal sinuses and nasal cavity, which only accounts for 0.5-4% of all sinonasal neoplasms with an incidence of 0.2-1.5 cases per 100,000 patients per year [5]. SNIP is commonly found in males, with an age group of the fifth to the sixth decade [1]. The majority of the patients have unilateral sinonasal tract involvement; however, there have been reported cases of bilateral involvement [6]. Our patient is a male and in his 60s presented with unilateral involvement.

An average percentage of malignant transformation of SNIP reported in the literature is 10% ranging from five to 27% [6}. Mirza et al. reported a 7.1% incidence of synchronous and 3.6% metachronous SCC in SNIP [7]. A concrete etiology for malignant transformation in SNIP is yet to be found. Retrospective analysis of 162 patients revealed an odds ratio of 12.7 for smokers to experience malignant transformation compared to non-smokers [8]. Our patient was a chronic smoker; hence our patient has a high risk of developing SNIP and potential malignant change.

It is challenging to differentiate SNIP and malignant transformation preoperatively, as there are no distinct clinical signs in-between these two [9]. Even histological diagnosis of malignant transformation of SNIP preoperatively is difficult as the biopsy may not include the area of SNIP with malignant change. Hence, multiple biopsies in the different locations of the tumor, especially near the base of the central area, to yield better results have been recommended [10]. But the majority of SNIP arises from the maxillary sinus; obtaining an office biopsy from this location is difficult in an awake patient [11].

Some radiological features help to differentiate SNIP from its malignant counterpart. Bony erosion and orbital wall involvement in CT imaging were suggestive of an aggressive tumor. However, orbital wall involvement was found in both recurrent tumors of SNIP and SNIP with SCC [11]. Jeon et al. reported in their case series of six patients that positron emission tomography (PET) cannot distinguish between SNIP and malignancy [12].

Magnetic resonance imaging (MRI) is superior to CT in distinguishing between SNIP and SNIP with SCC. SNIP demonstrated a unique pattern on MRI known as a convoluted cerebriform pattern (CCP), which is a mix of linear or curvilinear hyperintense and hypointense striations partially or diffusely seen in solid components of the tumor on T2-weighted or contrast-enhanced T1-weighted MRI [13]. Sixty percent of SNIP with SCC patients have a complete loss of this CCP compared to only 13% in SNIP alone in a larger study involving 35 SNIP with SCC patients and 31 SNIP alone patients by Yan et al. Furthermore, Yan et al. used diffusion-weighted imaging (DWI) sequences on MRI and measured apparent diffusion coefficient (ADC) value obtained from DWI [11]. El-Gerby et al. have achieved 90% accuracy and 100% sensitivity using an ADC value of 1.2 × 10 −3 mm2/s as a cut-off point to differentiate between benign and malignant sinonasal lesions; a low ADC value predicts malignant lesions [14]. Yan et al. combined loss of CCP and low ADC value and were able to predict malignant transformation by 100% specificity and 77.8% sensitivity in their study [11]. Thus it is recommended a combination of CT and MRI for SNIP patients to predict malignancy and offer appropriate surgical management.

Genetic factors such as muscle segment homeobox gene 2 (MSX2), programmed cell death 4 (PDCD4), Kirsten rat sarcoma virus gene (KRAS), and phosphatase and tensin homologue (PTEN) are postulated in malignant transformation. Proteins such as SCC antigen, fascin, survivin, cyclooxygenase-2 (​​​​​​​COX-2), cell adhesion molecules, and Ki-67 are found at higher levels in SNIP with SCC than in SNIP. However, a recent systematic review by Long et al. demonstrated that none of the aforementioned biomarkers conclusively differentiate SCC from SNIP currently [15]. 

Several other authors have also recommended a combination of CT, MRI, and PET/CT as a preoperative workup to differentiate SNIP from synchronous SNIP [16]. However, in developing countries like Malaysia, it is not practical to obtain CT, MRI, and PET/CT for all patients suspecting SNIP because of its cost and lack of facilities. In our patient, the extensive bony erosion and orbital wall involvement in the CT scan raised the suspicion of malignancy. 

An aggressive surgical approach to remove the tumor completely with postoperative radiation therapy is the recommended treatment for SNIP with SCC. Completeness of removal appears to be the key aspect, not the approach [17].

## Conclusions

A rapid increase in the size of a nasal swelling should raise a suspicion of malignant disease or malignant transformation of SNIP. CECT and MRI preoperatively can aid in an accurate diagnosis of synchronous SNIP with SCC from SNIP alone. Aggressive treatment of surgery and adjuvant therapy such as radiotherapy is required to prevent recurrence and to improve the overall survival rate.
